# Informing relatives about their hereditary or familial cancer risk: study protocol for a randomized controlled trial

**DOI:** 10.1186/1745-6215-15-86

**Published:** 2014-03-20

**Authors:** Eveline de Geus, Cora M Aalfs, Mathilde GE Verdam, Hanneke CJM de Haes, Ellen MA Smets

**Affiliations:** 1 Department of Medical Psychology, Academic Medical Centre, University of Amsterdam, Meibergdreef 9, 1105 AZ Amsterdam, The Netherlands; 2 Department of Clinical Genetics, Academic Medical Centre, University of Amsterdam, Meibergdreef 9, 1105 AZ Amsterdam, The Netherlands; 3 Research Institute of Child Development and Education, University of Amsterdam, Spui 21, 1012 WX Amsterdam, Netherlands

**Keywords:** Genetic counseling, Family communication, Hereditary cancer, Randomized controlled trial, Intervention

## Abstract

**Background:**

Genetic counseling for hereditary breast or colon cancer has implications for both counselees and their relatives. Although counselees are encouraged by genetic counselors to disclose genetic cancer risk information, they do not always share this information with their at-risk relatives. Reasons for not informing relatives may be generally categorized as a lack of knowledge, motivation and/or self-efficacy. Presented here is the protocol of a randomized controlled trial that aims to establish the effectiveness of an intervention focused on supporting counselees in their disclosure of genetic cancer information to their relatives.

**Methods/Design:**

A multicenter randomized controlled trial with parallel group design will be used to compare the effects of an additional telephone counseling session performed by psychosocial workers to enhance the disclosure of genetic cancer information to at-risk relatives (intervention group) with a control group of standard care. Consecutive index patients with relatives at risk for hereditary or familial breast and/or ovarian cancer or colon cancer, are randomly assigned (block size: 8; 1:1 allocation ratio) to the intervention (n = 132) or control group (n = 132, standard care). Primary outcomes are counselees’ knowledge, motivation and self-efficacy regarding informing their relatives.

**Discussion:**

This intervention may prove important in supporting counselees to disclose hereditary and/or familial cancer risk information to at-risk relatives and may enable more at-risk relatives to make a well-informed decision regarding genetic services and/or screening.

**Trial registration:**

This trial is registered in the Netherlands National Trial Register (NTR) with trial ID number NTR3745.

## Background

Identification of hereditary or familial breast or colorectal cancer risks has implications not only for counselees but also for their relatives. Knowledge of genetic test results in families is important to individualize recommendations for risk reduction for at-risk relatives (that is, DNA testing, regular breast or colon screening and/or (prophylactic) surgery) [1-3].

According to international [4] and Dutch [5] guidelines, genetic counselors encourage counselees to inform at-risk relatives about their genetic test result and the availability of surveillance options. Thereby, genetic counselors face the challenge of encouraging counselees to inform their relatives to enable them to make an informed decision about their own health, while also supporting counselees’ autonomy [6]. The counselees’ wish not to inform relatives has to be respected, and relatives’ wish not to know has to be taken into account since they may consider the information to be an invasion of their own privacy [7].

Although counselees report that they generally feel responsible to disclose genetic risk information to relatives [8-10], they do not always succeed in correctly informing all relevant relatives [11-15]. As a result, relatives may lack the opportunity to make a well-informed decision about whether or not to pursue genetic counseling, DNA testing and/or surveillance activities [4,16].

To date, several (mostly qualitative studies) have addressed counselees’ barriers to informing their relatives [14,15,17,18]. Barriers may be categorized as lack of knowledge, lack of motivation, and lack of self-efficacy. When counselees have a lack of knowledge, their understanding of family members that ought to be informed may not be sufficient [18,19]; moreover, insufficient knowledge may lead to incorrect disclosure [20]. A lack of motivation, may be due to the desire to protect the relative or oneself, for example, from negative reactions by the relative [10]. Moreover, counselees may consider a relative to be too emotionally fragile to burden them with genetic cancer information [19], or relatives may be perceived as not mature enough to understand the information [21] or as too old and, therefore, no longer in a high-risk life stage [14]. A lack of self-efficacy may lead to counselees feeling unable to inform their relatives. The counselee does not deliberately withhold information but may feel insecure about correctly disclosing the complex or burdensome information [22].

As a result of feeling responsible about the need to inform relatives, but at the same time facing several barriers, counselees may experience their messenger role as a burden [23]. Indeed, cancer genetic counselees report a need in genetic counseling for information and support in communicating the genetic information to others [24,25]. Counselees also express that their lack of confidence in disseminating information may be improved by more professional backup [7].

To address the need to support counselees and the call for an intervention by others [26,27], various interventions have been developed to improve family communication about genetic testing and screening options [28-31]. These interventions mainly comprise enhanced information [29,31] or communication skills training [28,31] for counselees. However, no effects on facilitating the family communication process were found, except for improving counselees’ satisfaction [31]. One study developed a counseling intervention to support counselees in communicating genetic information to at-risk relatives; this intervention comprised the use of a pedigree chart (for example, a document to record medical family history) to explicitly identify at-risk relatives, a follow-up letter stressing the importance of family disclosure, and two follow-up telephone calls during which it was documented which relatives had been informed and offering guidance about how to approach relatives [32]. Investigation of the effectiveness of this intervention indicated that the proportion of at-risk relatives using genetic services was larger in the intervention group (61%) compared with the control group (36%); however, this latter study had a cohort design (instead of a randomized design) thereby limiting the possibility to draw firm conclusions [32].

This paper describes the design of a randomized controlled trial (RCT) for investigating the effectiveness of an intervention based on the principles of Motivational Interviewing (MI) [31]. MI is a client-centered counseling style that helps individuals change health-related behavior, such as sharing hereditary cancer risk information with relatives. We consider that this client-centered but directive counseling style of MI is well suited for counselors, since they have to discuss family communication with counselees while at the same time respecting counselees’ autonomy.

The intervention, consisting of an additional telephone counseling session conducted by psychosocial workers, aims to support counselees in disclosing hereditary or familial cancer risk information to at-risk relatives. This paper presents details of the design of this RCT, guided by the CONSORT checklist [33].

This RCT (registered in the Dutch National Trial Register: NTR3745) aims to test the following hypotheses:

**Hypothesis 1** - The intervention will increase counselees’ knowledge, motivation and self-efficacy with regard to informing relatives about their hereditary or familial cancer risk, as compared with counselees who receive standard care.

**Hypothesis 2** - The intervention will lead to more at-risk relatives being informed by counselees; increased knowledge among relatives about hereditary/familial cancer and preventive measures; and increased intention of informed relatives to engage in genetic counseling, testing and/or preventive measures, compared with the standard care condition.

**Hypothesis 3** - The intervention will be positively evaluated by counselees and psychosocial workers.

## Methods/Design

### Trial design

The study comprises a multicenter RCT with a parallel group design, comparing the effects of an additional telephonic counseling session aimed at supporting counselees in informing their at-risk relatives (intervention group), with a control condition of standard care only. Participating counselees will complete a questionnaire at three moments:

1. Pre-intervention (T1), after receiving a summary letter from the genetic counselor,

2. Post-intervention (T2), immediately following the intervention, or -in standard care - at the same time (about 4 weeks after T1),

3. Follow-up (T3) at 4 months after T1.

**Figure 1:**
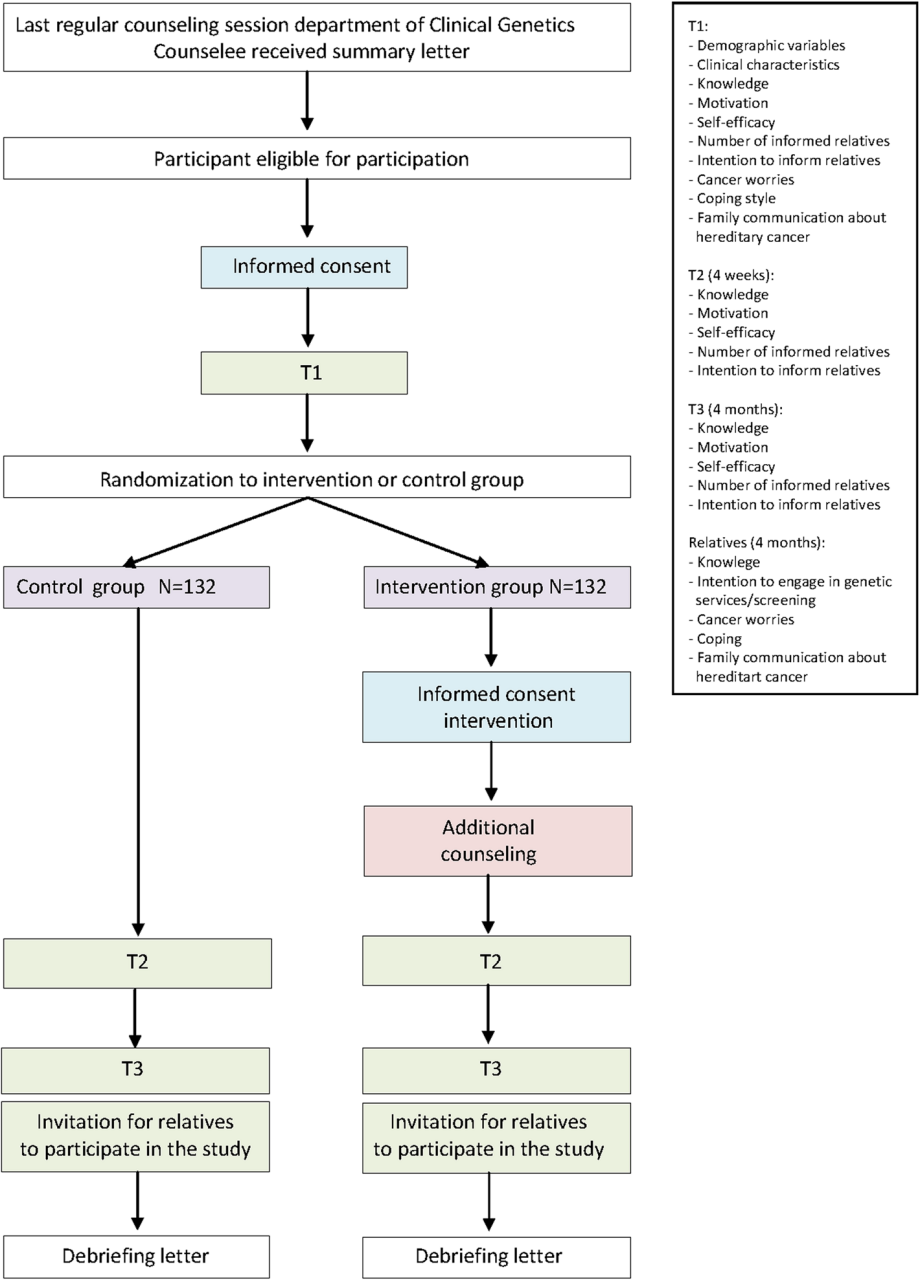
Flowchart of the randomization procedure, intervention and data collection.

At T2 counselees are telephoned to complete items regarding their insight as to who to inform and their knowledge on surveillance measures; the rationale behind the telephone intervention is that these items are too complex to complete alone. At T3, participants will invite at-risk relatives to also complete a questionnaire (Figure 1).

### Participants

Consecutive counselees visiting the department of Clinical Genetics of three university hospitals in the Netherlands will be recruited. The sample comprises the following groups of counselees: those with conclusive test results (a pathogenic mutation has been found), those with an inconclusive test result, and those in whom DNA testing is not possible. For the latter two groups there is an increased cancer risk based on family history.

#### Inclusion criteria

Participants will be counselees who: 1) are the first in their family to visit the Clinical Genetics department for hereditary or familial colon or breast and/or ovarian cancer (referred to as: index patients); 2) have at least one relative at increased risk, that is, eligible for genetic testing and/or surveillance; 3) are aged ≥ 18 years, and 4) are able to read/write Dutch.

#### Relatives

At T3, the first, second and third-degree relatives are invited by the counselee to complete a single questionnaire. Eligible relatives are at increased risk (that is, eligible for genetic testing and/or regular surveillance), are aged ≥18 years, and able to read and write Dutch.

### Recruitment and informed consent

#### Counselees

In the Netherlands, it is considered standard care for the Clinical Genetics department to send counselees a summary letter after the last counseling session; this letter reaffirms the information provided and aims to facilitate the communication process in the family. When this letter has been received, clinical geneticists and counselors will invite eligible counselees to participate using an information letter, an informed consent form, a return slip to decline participation, and an invitation to complete a (web-based) questionnaire (T1).

We will use a two-stage randomization and a consent procedure with consent to postponed information [34-36]. In this procedure, counselees are masked for the exact study objective. However, they are informed that: 1) the study aims to assess issues pertaining to family disclosure, 2) the study has an additional purpose, and 3) a letter with the postponed information about the additional research question and reasons for not informing them during recruitment will be sent after the study. Postponed information enables a blind design and avoids a less favorable outcome in the control group resulting from their knowledge that the other counselees receive extra care. This procedure has been used previously and resulted in no major objections [35-38].

In case of consent, counselees will be asked to complete a web-based baseline questionnaire (if preferred, a paper version of the questionnaire is available). Counselees will then be randomized (Figure 1). Counselees in the intervention group will receive an information letter about the additional counseling, an informed consent form for the intervention, and a return slip to decline participation. Within one week they will be contacted by telephone to provide them with additional information about the intervention and are asked for their consent. Counselees in the control group will receive no additional information. Information about the randomization procedure will be included in the debriefing letter for counselees, which is sent after the study.

#### Relatives

In the Netherlands, medical-ethical regulations preclude counselors/researchers from directly approaching a counselee’s relatives. Therefore, at T3 all counselees will be asked to inform their relatives about the study. In case of consent, counselees will receive more detailed information and the family pedigree will be used to decide which individuals are eligible for study inclusion. Then, counselees will be asked to invite (by mail) their eligible first, second and third-degree relatives to participate in the study. For this, they can use an introductory letter, a questionnaire and a return envelope, which are made available by the researchers. Relatives willing to participate complete the questionnaire, thereby giving their consent, and return the questionnaire to the researchers. Researchers do not collect the full names/addresses of the relatives but assign a study ID number to each relative. In case of nonresponse, counselees will be asked to send their relatives a reminder after three weeks.

### Control group

Counselees in the control group will receive standard care only. It is recommended by the Dutch Society for Clinical Genetics that, during the first consultation, counselees are informed about the possible consequences of genetic testing for their relatives [5]. If the genetic counseling result is relevant for relatives, counselees are asked to inform their at-risk relatives. After the last counseling session, counselees receive a summary letter which reaffirms the information provided during the counseling. In case of hereditary cancer, a separate family letter is provided about the test results and recommendations for surveillance measures for the counselees and their relatives. In the Netherlands, although all departments of Genetics consider providing these letters as part of standard care, no specific guidelines are available; this implies that some differences may exist between these letters.

### Intervention group

Counselees in the intervention group will receive an additional counseling session provided by psychosocial workers specialized in genetics, aimed at supporting them to inform their at-risk relatives. The intervention will be delivered by telephone. The rationale for this method is that: 1) this allows for contact with counselees with limited knowledge/motivation to inform relatives and who are therefore unlikely to visit the department for additional counseling, 2) a telephone intervention is more likely to be implemented in daily care, and 3) a telephone-based intervention was earlier found to be effective in increasing the number of relatives that attended genetic services [32].

We developed a five-step model for the additional counseling session [39] (Table 1) based, in part, on the work of Rollnick *et al*. [40]. Their directive client-centered counseling style, known as Motivational Interviewing (MI), elicits behavior change by helping clients to explore and resolve ambivalence [40]. MI is guided by the following principles: expressing empathy by use of reflective listening, developing discrepancy between goals and current behavior, assuming that clients are responsible for the decision to act or not, rolling with resistance rather than confronting, and supporting self-efficacy.

**Table 1:** Intervention elements

**Phase 1: exploring**		
Step 1	Agenda setting	Introducing the subject of family communication about hereditary or familial cancer risks and preventive measures. Explicitly ask the counselee if he/she is willing to discuss this issue.
Step 2	Exploring	Systematically exploring counselees’ current and planned pattern of informing relatives. Psychosocial workers investigate whether the counselee correctly knows which family members are at risk and which information should be provided to them.
*Evaluation*		*After the first phase the psychosocial worker verifies whether or not all at-risk relatives are informed thoroughly (thoroughly = at least the written advice in the summary letter). If the counselee has informed all at-risk relatives properly the psychosocial worker will end the counseling session. If not, the psychosocial worker will start phase 2.*
**Phase 2: additional information and brainstorming**		
Step 3	Additional information	Additional or corrective information is provided, if needed. For example, in case of BRCA 1/2 it is important to stress that male relatives can also transmit the risk and thus need to be informed.
Step 4	Building motivation	The purpose of the fourth phase is to build motivation and strengthen self-efficacy. Psychosocial workers invite counselees to verbalize arguments in favor of informing relatives, to reinforce these arguments and thus strengthen the counselees’ motivation. Any barriers are also assessed and discussed. These barriers can be practical, for example, not having personal contact, and/or emotional, for example, causing too much distress. Also, reasons for (non)disclosure are explored, so as to understand the counselee’s perspective and motives, to build rapport and reduce resistance. Likewise, the counselees’ confidence in their ability to correctly and sufficiently inform relatives is assessed and strengthened.
Step 5	Brainstorming	During the final phase, the psychosocial worker invites the counselee to engage in active brainstorming on possible solutions for informing more difficult to approach relatives. The goal is to develop a plan for informing these relatives.

The intervention is delivered by one of five psychosocial workers. All will have attended two days of training days provided by two trainers from the department of Medical Psychology (AMC) who have extensive experience with MI. These sessions provide more information about MI and the intervention. Moreover, interviewing techniques are practiced with a trained actor and individual feedback is provided by the trainers.

As the psychosocial workers have access to counselees’ medical files and also have their pedigree available they are well informed; this avoids counselees having to discuss their situation/issues again. Counselees are informed about this procedure as part of the informed consent procedure.

Details on the development of the actual intervention will be published separately.

### Quality of the intervention

All intervention sessions will be audio-recorded to: examine whether the psychosocial workers perform their task according to the intervention protocol, trace differences between the psychosocial workers, and monitor changes in intervention delivery over time.

All recordings will be divided into four sets of 33 consecutive sessions. From each set 12 recordings will be chosen, stratified by psychosocial worker, and analyzed to investigate whether psychosocial workers correctly follow the steps of the intervention (Table 1). Scoring of the 48 recordings will be based on an adapted version of an instrument developed earlier [41].

### Primary outcome measures

At T2 the primary outcomes will be assessed by questionnaire (and partly by telephone) using specific instruments (Figure 1). Details on the development and psychometric properties of the outcome measures will be published separately.

Knowledge will comprise counselees’:

1. Insight into which relatives need to be informed, as indicated by a percentage ‘correct knowledge’ specifying the relative number of family members that need to be informed, correctly remembered by the counselee (range 0 to 100).

2. Knowledge about surveillance measures for relatives, as indicated by the percentage ‘correct knowledge about surveillance options’ that indicates how many screening options for relatives (1 to 6), are correctly remembered by the counselee (range 0 to 100).

3. Risk perception, as measured with two items: perceived lifetime breast and/or ovarian or colon cancer risk in percentages, and perceived breast and/or ovarian or colon cancer risk as compared with the average Dutch woman or person (5-point Likert scale: 1 = very strongly lowered, 5 = very strongly heightened).

4. General knowledge about hereditary breast or colon cancer, which includes six items developed by Pieterse *et al*. [42] based on the work of Claes *et al*. [14]. All items are presented as statements with three response categories (‘Correct’, ‘Incorrect’, or ‘I do not know’). A higher score indicates more knowledge about hereditary cancer.

#### Motivation

Counselees’ motivation to inform relatives will be assessed using a 30-item instrument based on a 36-item questionnaire developed by Finlay *et al*. [43]. All items are potential determinants of disclosure of information to relatives and are answered on a 5-point Likert scale (1 = plays no role in disclosure, 5 = plays a large role in disclosure). Items will be presented to participants as: ‘Reasons to inform relatives’ (positive motivation, 13 items) and ‘Reasons for not informing relatives’ (negative motivation, 17 items). For each scale a total score is created, with a higher score indicating more positive or negative motivation to inform relatives (range 13 to 65 and 17 to 85, respectively).

#### Self-efficacy

Self-efficacy is the measure of one’s own ability to complete tasks and reach goals [44]. Index patients will be asked to indicate how sure they are that they are able to overcome seven obstacles in informing relatives, on a 4-point Likert scale (1 = not sure at all, 4 = very sure). A total score is created, with a higher score indicating more self-efficacy (range 7 to 28).

### Secondary outcome measures

Secondary outcome measures include the following:

1. Relatives’ knowledge, which will be assessed using the same instruments as used by counselees, comprising knowledge about surveillance, risk perception and general knowledge about hereditary breast and/or ovarian cancer or colon cancer.

2. Relatives’ intention to engage in genetic counseling, testing and/or preventive measures, measured with six items using the following format ‘How likely is it that you will have a colonoscopy in the coming year?’ Items are scored as 1 = very unlikely, to 5 = very likely [45].

3. Relatives’ evaluation of the way they have been informed, which includes seven items about the way relatives are informed and their evaluation of the communication process. Items were developed for the purpose of this study and are based on those developed by Vos *et al*. [46].

4. Counselees’ evaluation of the intervention, which includes 18 questions about the intervention and its content, psychosocial workers’ performance, and practical issues related to the intervention (for example, right moment in time, intervention conducted by telephone).

5. Psychosocial workers’ evaluation of the intervention, in which psychosocial workers are asked to complete ten items after each additional counseling session to evaluate the intervention. The aim is to gain insight into their experiences with the intervention, to identify issues to improve the intervention, and to track (in) congruence between counselees’ and psychosocial workers’ experiences with the intervention.

### Counselees’ characteristics

Counselees’ characteristics include the following:

1. Demographic variables, which include age, gender, educational level, age of their children, and marital status.

2. Clinical characteristics, comprising genetic test result, cancer diagnosis, number of relatives to be informed, number of informed relatives (as reported by the counselee), age at which the counselee was first confronted with cancer in the family, and types of cancer in the family.

### Sample size

For the primary research question we aim for a medium effect size. At baseline (T1), we assume no differences in primary outcomes between the intervention and control group. At T2 we expect to find a medium effect size. We assume this to be 0.5 for the intervention group and 0.2 for the control group, taking into account that the outcomes in the control group may be slightly affected by that fact that they are aware that the study focuses on informing their relatives; this effect is estimated to last until follow-up (T3).

Based on these assumptions, we require 132 patients in each group to have 80% power to detect an intervention effect (group/time interaction) that is significant at the 0.05 level (assuming a 0.7 correlation between measurements). In addition, we have a 99% chance to detect differences between measurement moments and a 46% chance to detect group differences overall.

Assuming a response rate of 50% and a 20% dropout, we need to approach 660 counselees. Preliminary work has shown that counselees are advised to inform (on average) 4.6 at-risk relatives. Assuming that counselees provide contact information for (on average) two relatives and that 50% of these relatives will complete the questionnaire, it is estimated that we will have data on 264 relatives.

### Randomization

ALEA (a software package to support online randomization in healthcare research) will be used to randomly allocate the participant to either the intervention or control group (allocation rate 1:1). This computer-based system ensures allocation concealment. In order to create equal comparison groups and prevent allocation bias, randomization will be stratified for three different groups (that is, conclusive versus inconclusive DNA test result) and whether or not the counselee has been diagnosed with cancer. To balance group sizes, random block randomization will be used with a maximum block size of 8 and block size factor 5.

### Blinding

The informed consent procedure to postpone information enables a blind design for participants in the control group. Participants in the intervention group will be masked for the exact study objective to avoid biased outcome measures. Psychosocial workers and research assistants who collect data cannot be blinded. At T2, research assistants will obtain outcome measures by telephone. To ensure that the outcomes are obtained as objectively as possible, a telephone script has been developed to minimize individual differences between research assistants. Also, to minimize additional bias, data entry is performed by someone other than the person who performs the analyses.

### Data analysis

All analyses will be based on the intention-to-treat principle. Assuming random dropout, incomplete data will be imputed using data of the control group, that is, standard care.

*Hypothesis 1:* Multilevel analysis will be used to investigate whether the intervention increases counselees’ knowledge, motivation and self-efficacy as compared with the control group. Effects of the intervention will be investigated on each of the seven outcome variables separately. The three moments of measurement (T1, T2, T3) will be treated as nested within counselees.

*Hypothesis 2:* Multilevel analysis will also be used to investigate whether the intervention increases: the number of correctly informed relatives; relatives’ knowledge about hereditary/familial cancer and preventive measures; and relatives’ intention to engage in genetic counseling. For this, the relatives will be treated as nested within counselees and counselees will be treated as nested within physicians.

*Hypothesis 3:* To investigate how counselees and psychosocial workers evaluate the intervention, descriptives and frequencies will be calculated to present counselees’ and psychosocial workers’ evaluation of the intervention.

### Ethical and safety issues

The study has been approved by the Medical Ethics Committee of AMC (MEC 2012-02) and recruitment of the participants is in progress. The study is conducted according to the principles of the Declaration of Helsinki and in accordance with the Medical Research Involving Human Subjects Act. After participants have given informed consent, each participant receives an individual ID number to secure anonymity. The web-based questionnaires can only be completed by participants with this unique number and data from these questionnaires are collected on a secured server. In accordance with the act, the investigator will inform the participants and the accredited Medical Ethics Committee if anything occurs from which it appears that the disadvantages of participation may be significantly greater than was foreseen in the research proposal.

## Discussion

A multicenter RCT is proposed to support counselees in disclosing hereditary or familial cancer risk information to at-risk relatives. The aim of the intervention is to evaluate the effectiveness of an additional telephonic counseling session performed by psychosocial workers.

Genetic counselors rely on counselees to inform their relatives about their hereditary or familial cancer risk and the possibilities to reduce this risk. Therefore, it is important to maximize their ability to be a competent, motivated and confident informant while respecting their wish not to inform (some) relatives. The proposed intervention aims to support counselees in this difficult task and hopefully allow more at-risk relatives to make a well- informed decision. If effective, this intervention might lead to more relatives taking up genetic services and preventive screening and possibly reducing cancer morbidity and mortality in these affected families.

This is not the first intervention to enhance family communication about genetic testing and hereditary risk information [28-30]. However, to our knowledge, this is the first to 1) study the effectiveness of additional support using a randomized controlled design based on MI, 2) apply an intervention for mutation carriers and counselees with relatives with an increased risk to develop cancer, and 3) involve relatives in the study. Another strength is that the psychosocial workers delivering the intervention are specialized in genetics, are experts in working with cancer counselees, have well-developed communication skills, and are likely to have more time to deliver the intervention than clinical geneticists and/or genetic counselors.

Some limitations also need to be addressed. First, our patient sample is not truly representative for cancer counselees in general, since only index patients are invited to participate; this may affect the external validity. We did consider inviting all counselees who were advised to inform at-risk relatives, but rejected this idea because of the complexity of establishing which relatives have already attended genetic services and/or have already been informed by other relatives about their risk to develop cancer.

Second, similarity of the two study arms is not guaranteed since participants in the intervention arm receive more attention than the controls. Therefore, it is difficult to precisely define the effective components of the intervention. Also, a third study arm, comprising a condition with only a telephone call was considered to be too ambitious given the additional number participants required. However, to gain insight into the effective components of the intervention, we aim to compare the group of counselees who only needed the first phase of the intervention (that is, systematically exploring disclosure of genetic cancer information to at-risk relatives) with those who also received the second phase of the intervention (that is, building motivation, self-efficacy, brainstorming).

Moreover, pilot work revealed the complexity of gaining reliable insight into the counselees’ knowledge regarding which relatives to inform and what to tell relatives by asking them to complete questionnaires. Therefore, completion of the questionnaire after the intervention (T2) is done (in part) by telephone to ensure that the counselees understand the items correctly. This telephone call, performed by the researchers, might be seen as an intervention on its own, since counselees are asked about family communication in a systematic way. For that reason, the telephone call is scripted and standardized as far as possible.

Finally, studies in which relatives are invited to participate, including the present study, have some limitations. Medical-ethical regulations preclude geneticist/researchers from directly approaching the relatives of counselees. Therefore, we ask the participating counselees to invite their relatives to participate; this may cause some selection bias since only those relatives can be invited who have been correctly informed by the counselee. To prevent this bias, we use the pedigree of the participant, and for each individual relative, we systematically ask whether this relative may be invited or not.

This paper is the first of a series that will outline the development and testing of an intervention to enhance family communication about hereditary or familial cancer risks; here we describe the study protocol of the RCT. Additional papers will describe the development and psychometric properties of the outcome measures, pilot testing, and the outcomes of the trial.

## Trial status

A pilot study has been performed and recruitment for the trial is in progress. Data collection will continue until summer 2014.

## Abbreviations

MI: motivational interviewing; RCT: randomized controlled trial.

## Competing interests

The authors declare that they have no competing interests.

## Authors’ contributions

EDG drafted the paper. ES and CA are the study’s principal investigators and participated together with HDH in the design of the study. MV assisted with the data analyses. All authors made substantial contributions to the design of the study and to critical revision of the paper; all authors read and approved the final manuscript.

## Authors’ information

E. de Geus, MSc, psychologist, PhD student, Department of Medical Psychology, Academic Medical Centre, University of Amsterdam, the Netherlands. C.M. Aalfs, PhD, clinical geneticist, Department of Clinical Genetics, Academic Medical Centre, University of Amsterdam, the Netherlands. M.G.E. Verdam, MSc, psychologist and statistician, Department of Medical Psychology, Academic Medical Centre, University of Amsterdam, the Netherlands and Research Institute of Child Development and Education, University of Amsterdam, The Netherlands. J.C.J.M. de Haes, PhD, psychologist, head of the Department of Medical Psychology, Academic Medical Centre, University of Amsterdam, the Netherlands. E.M.A. Smets, PhD, psychologist, Department of Medical Psychology, Academic Medical Centre, University of Amsterdam, the Netherlands.
